# Conventional oil and natural gas infrastructure increases brown-headed cowbird (*Molothrus ater*) relative abundance and parasitism in mixed-grass prairie

**DOI:** 10.1098/rsos.170036

**Published:** 2017-07-12

**Authors:** Jacy Bernath-Plaisted, Heather Nenninger, Nicola Koper

**Affiliations:** Natural Resources Institute, University of Manitoba, 303-70 Dysart Road, Winnipeg, Manitoba, Canada R3T 2M6

**Keywords:** brood parasitism, energy development, oil and gas infrastructure, grassland songbirds, edge effects, nesting success

## Abstract

The rapid expansion of oil and natural gas development across the Northern Great Plains has contributed to habitat fragmentation, which may facilitate brood parasitism of ground-nesting grassland songbird nests by brown-headed cowbirds (*Molothrus ater*), an obligate brood parasite, through the introduction of perches and anthropogenic edges. We tested this hypothesis by measuring brown-headed cowbird relative abundance and brood parasitism rates of Savannah sparrow (*Passerculus sandwichensis*) nests in relation to the presence of infrastructure features and proximity to potential perches and edge habitat. The presence of oil and natural gas infrastructure increased brown-headed cowbird relative abundance by a magnitude of four times, which resulted in four times greater brood parasitism rates at infrastructure sites. While the presence of infrastructure and the proximity to roads were influential in predicting brood parasitism rates, the proximity of perch sites was not. This suggests that brood parasitism associated with oil and natural gas infrastructure may result in additional pressures that reduce productivity of this declining grassland songbird.

## 1. Introduction

As a group, population sizes of North American grassland songbirds are declining precipitously, with 75% of species displaying negative population trends between the years of 1966 and 2013 [1]. Grassland songbirds currently face a multitude of threats, primarily from habitat loss and degradation (e.g. [2,3]), and identifying the mechanisms that explain effects of fragmentation is necessary to identify suitable mitigation strategies. The spread of energy infrastructure across the Northern Great Plains may contribute to some population declines among grassland songbirds [3,4]. Over the past century, Canada's mixed-grass prairie ecoregion has experienced a dramatic increase in the density of conventional oil and natural gas extraction infrastructure (e.g. [4–6]). Since 1900, over 300 000 conventional oil and natural gas wells have been drilled in Alberta [5], and currently, there are approximately 5000 natural gas compressor stations in this province [7]. Annually, nearly 35 000 ha of Canada's mixed-grass prairies are disturbed by oil and gas activities [8]. The growing presence of industrial structures and associated linear features, such as access roads, power transmission and distribution lines, and fences, across the prairie interior has the potential to introduce edges and increase perch availability for predators or brood parasites in this otherwise relatively planar system.

Increased density of edge habitat has been linked to both increases in brown-headed cowbird (*Molothrus ater*; hereafter, ‘cowbird’) density [9] and brood parasitism rates (e.g. [10–12]). Similarly, there is evidence that cowbirds prefer habitat with high perch availability [13] and brood parasitism rates may be higher near potential perch sites [14]. However, these patterns are less well understood across mixed-grass prairie ecosystems exposed to industrial development. If energy development in the Great Plains does indeed facilitate cowbird brood parasitism, it may place an additional stressor on grassland nesting songbird species in this region. Increased cowbird abundance and brood parasitism may lead to declines in avian host species, as cowbird brood parasitism negatively affects host population fecundity and growth (e.g. [15–17]) through nest abandonment [18], reduction in the number of host young fledged [17,19,20] and greater risk of predation [21]. Further, adult cowbirds are documented nest predators [22,23] and will destroy eggs and kill nestlings [24].

Cowbird brood parasitism is well documented in grassland songbirds, though often at relatively low frequencies compared with brood parasitism rates in forested ecosystems (e.g. [20,25,26]). However, abundances of cowbirds in grasslands influenced by industrial development might be elevated. The brown-headed cowbird is an obligate brood parasite that evolved on the Great Plains, where it is hypothesized that it depended on American bison (*Bison bison*) herds and the insects associated with bison for subsistence; currently, cowbirds are known to parasitize the nests of over 220 host species [27]. The range of the brown-headed cowbird has greatly expanded over the past 200 years, as cowbirds have benefited from many anthropogenic alterations to the landscape, including agricultural land conversion and increasing habitat fragmentation [10,20]. Relative to other ground-nesting songbird species breeding in the northern mixed-grass prairie ecosystem, Savannah sparrow (*Passerculus sandwichensis*) is among the most frequent cowbird hosts [28–30] with brood parasitism rates approaching 30% of nests in some regions [28,31]. However, it should be noted that brood parasitism rates for this species vary greatly across its wide distribution [25,32]. In Canada, the Savannah sparrow population is currently decreasing by 1.3% annually [1]. Because we have demonstrated elsewhere that Savannah sparrow abundance is independent of distance to oil wells in this region [33], Savannah sparrows were also useful as a focal species for our study as nests were present at a range of distances from wells, allowing us to evaluate effects of distance to wells on brood parasitism rates.

We investigated two hypotheses regarding the effects of oil wells and natural gas compressor stations on cowbird relative abundance (an unadjusted index of density) and brood parasitism rates of Savannah sparrow nests in Alberta's mixed-grass prairie. We focused our study design around oil wells and compressor stations, which have the largest above-ground footprints associated with non-renewable resource extraction in this region. First, we hypothesized that cowbirds may perceive oil and gas infrastructure and associated features as edges and may use structures and fences as perches. Therefore, we predicted that infrastructure would have an overall positive effect on cowbird relative abundance. If cowbirds are attracted to edges or perch availability, we predicted that cowbird relative abundance would be higher at sites with infrastructure relative to controls. Similarly, within sites, we expected that cowbird relative abundance would increase with proximity to anthropogenic features. Further, we predicted that this increase would lead to higher brood parasitism rates of Savannah sparrow nests at infrastructure sites and in close proximity to structures. Finally, it has been hypothesized that cowbirds use perches to enhance their ability to find host nests [34–36]. Thus, we predicted that brood parasitism frequency would be positively correlated with proximity to perches within sites.

## 2. Methods

### 2.1. Study area

The approximate centre of our study area was located in Brooks, Alberta (50°33′51^″^ N, 111°53′56^″^ W), with all study sites located within a 50 km radius of Brooks. Brooks is located at an elevation of 758 m above sea level and receives an average annual precipitation of 348 mm [37]. The landscape surrounding Brooks is 61% grasslands, which comprise both native mixed-grass prairie [38] and, to a smaller extent, exotic herbaceous grasslands. The remaining land area in the region is occupied primarily by cropland and small amounts (less than 5%) of water [39]. Predominant native plant species include blue grama (*Bouteloua gracilis*), needle-and-thread (*Hesperostipa comata*), western wheatgrass (*Pascopyrum smithii*) and junegrass (*Koeleria macrantha*); goatsbeard (*Tragopogon dubius*) and crested wheatgrass (*Agropyron cristatum*) are two of the most common exotic plant species in the region, but occurred in very low abundances in our sites. Sites averaged 7% exotic vegetation, and 42% of sites contained some exotic vegetation. Our inferences are thus primarily restricted to effects of wells in native prairies. All sites were grazed by cow–calf pairs at stocking rates ranging from 0.11 to 1.29 AUM ha^−1^. AUM is defined as Animal Unit Month, one mature cow and her suckling calf weighing a cumulative 1000 pounds (a 920-pound cow with an 80-pound calf) requiring 26 pounds of dry matter (DM) forage per day [40]. We did not include cattle stocking rates in our analyses here because elsewhere we have demonstrated that brown-headed cowbird densities are independent of cattle stocking rates in Canadian mixed-grass prairies at the field scale [41,42], probably because cowbird home ranges are very large and extend beyond the borders of individual fields [43]. Brooks is located in a region of high conventional oil and natural gas extraction activity; average oil well and natural gas well densities in this area are 0.5 km^−2^ and 5.9 km^−2^, respectively. The Brooks area is also located in a region of high brown-headed cowbird density [1].

#### 2.1.1. Sites

We surveyed the relative abundance of brown-headed cowbirds, and nesting success of Savannah sparrows, within 48 sites, each of which was defined by an 800 × 200 m (16 ha) survey area. Each site was located in a different 64.7 ha quarter section (approx. 800 × 800 m land planning unit), which is approximately the average size of a brown-headed cowbird breeding home range [43]. Each site contained an infrastructure lease or a control point at its centre. Each site was located on a flat to gently rolling mixed-grass prairie quarter section that was itself surrounded by other mixed-grass prairie patches on all four sides to minimize effects of crop edges. We sampled at 34 infrastructure sites and 14 controls. Control sites consisted of open mixed-grass prairie and did not contain oil wells or compressor stations. Infrastructure sites contained a pumpjack (*n* = 13), screwpump (*n* = 16) or natural gas compressor station (*n* = 5). Both pumpjacks and screwpumps are surface structures designed to extract oil from beneath the ground, though they do so by different mechanisms [44]. Pumpjacks (7 m) are taller in vertical structure than screwpumps (2–3 m). Both pumpjacks and screwpumps may be powered by propane generators or the local electric grid, and in the latter case, they may be associated with power distribution lines. Natural gas compressor stations are large structures that serve to pressurize pipelines used in the transport of natural gas; they contain multiple motors, turbines and sheds of varying sizes [44]. Each oil well or compressor station lease in the Brooks region disturbed an area ranging from 258 to 4986 m^2^. Lease sites at all infrastructure types included some bare ground, technical or barbed wire fences, and dirt-packed or gravel access roads.

Sites were orientated either east–west or north–south and avoided obstacles such as dirt berms and construction dugouts providing water for livestock. We chose a long, rectangular shape for our sites to capture a range of distances from focal infrastructure. Centre points were at least 400 m away from one another, and centre points of control sites were at least 800 m away from any oil infrastructure or natural gas compressor stations. The distance from the edge of a site to the nearest edge of another site ranged from a minimum of 50 m (nest plots—entire site) or 280 m (abundance transects—100 × 800 m at site centre), to a maximum of 15 km. We minimized the likelihood that individual cowbirds were recorded at more than one site during abundance surveys by selecting sites that were distant from other sites when possible (this included not doing abundance surveys at two nest-searching sites that were close to other sites); by surveying prior to 16.00 Universal Coordinated Time (UTC, which is 6 h ahead of the time zone of this study), when movement of cowbirds is relatively local [43]; by conducting surveys at adjacent sites concurrently or during the same morning, during this period of lower translocation; and by sampling relative abundance within only the 50 m closest to each transect centre, so that abundance transects were narrower. Nonetheless, we recognize that we could not eliminate the possibility of cowbirds occasionally moving among a few of our sites.

At each site, we used GPS units (Garmin eTrex 10) to map the locations of focal infrastructure and other anthropogenic features, such as fences, roads, power transmission and distribution lines, and natural gas wellheads. Natural gas wells consisted of an inverted U-shaped pipe emerging from a grass-covered base, surrounded by technical fencing to exclude cattle; the footprint of the pipe and fencing around each natural gas well averaged 23.1 m^2^ and had a height of 1.44 m. Natural gas wells were not central to our study design, as their footprints average only 0.3% of the footprint of an oil well, and they are silent and are not associated with permanent roads or human activity [45]; thus, their ecological impact was assumed to be small in comparison with effects of oil wells and compressor stations. Nonetheless, we included their presence in our analyses as potential perch sites in case they did contribute to changes in relative abundance of brown-headed cowbirds.

We defined roads as hard-packed dirt and gravel surfaces, excluding grass two-tracks or trails in this category, which were relatively uncommon at these sites. The majority of roads mapped in the study were county range and township roads or oil-access roads. We limited confounding landscape variables by selecting sites that were in areas without paved roads, wetlands, trees or steep topography.

### 2.2. Data collection

#### 2.2.1. Cowbird relative abundance transects

We measured brown-headed cowbird relative abundance at each site along 100 × 800 m transects centred within each site. In both 2013 and 2014, two rounds of transects were conducted beginning in the last week of May and concluding by 1 July, between dawn and 16.00 UTC, in conditions with sustained winds less than 20 km h^−1^ and little to no precipitation or fog. The observers conducted surveys by walking down the centre line of each transect and recording brown-headed cowbird abundance, regardless of sex, heard or seen within 50 m of the observer. It took 80 min to complete one transect, as this was the length of time it took us to slowly walk 800 m while identifying all birds seen or heard. When cowbirds were detected, we recorded observer location on the transect line, the bird(s)'s distance from observer, and the bird(s)'s bearing, to allow us to calculate the Universal Transverse Mercator (UTM) coordinates for each bird or group of birds observed.

#### 2.2.2. Nest searching and monitoring

Nest searching and nest vegetation sampling in this study were conducted at the same study sites and according to the same protocols described in Bernath-Plaisted & Koper [46]. We collected data on nesting success, number of young fledged and parasitism rates in breeding Savannah sparrows in both 2013 and 2014. Nest searching was conducted throughout the entirety of the same 800 × 200 m study sites at which cowbird relative abundance was sampled. Because grassland songbird nests are difficult to locate using the behaviour of adult birds, which are cryptic and secretive, we searched for nests using rope drags [47]. In both years, we conducted three rounds of rope-drag searches over the duration of the breeding season starting in the last week of May and concluding by the end of the third week of July. We conducted rope-drag searches each day as soon as the grass was dry (i.e. between 12.30 and 14.00 UTC) and surveyed no later than 18.00 UTC each day. We limited our nest-searching efforts to this time frame because research suggests that grassland songbirds are most likely to be on the nest early in the day [48].

We marked nests with bamboo stakes 10 m to the south and high-visibility pink chasers secured in the ground with nails 10 m to the west, took UTM coordinates and photographs of nest contents, and recorded the presence of brown-headed cowbird eggs and nestlings and host eggs and nestlings. Photographs were used to help confirm host species identity in cases when the adult was not observed. We returned to monitor nests at 2–5 day intervals for the duration of nest activity, visiting more frequently (2–3 day) when nests were near fledging age. At nest checks, we also photographed nest contents, aged nestlings, and recorded the presence of adult birds and any evidence of parasitism, depredation or abandonment. To improve our confidence in assigning nest fates, we also performed 30 min fledgling searches on empty nests that had contained nestlings within a reasonable age of fledging on the previous visit (i.e. nestling that would be 8 days or older on current visit). During fledge checks, we observed adults for fledgling provisioning activities, gauged defensive behaviour of adults and searched the immediate area for fledglings. We considered nests successful if they fledged host young and failed if they did not. We estimated the number of host young fledged by the number of nestlings known to be in the nest on the penultimate nest check (i.e. the last check before fledging occurred). Finally, upon the termination of each nest, we conducted vegetation sampling in a 1 m^2^ plot with the nest at the centre. Vegetation plots were established by placing two metre sticks so that they intersected across the centre of the nest to create four sampling quadrats. We used a Wien's pole [49] to measure stem height, density and litter depth, and we estimated the per cent cover of grasses, forbs, lichens, moss, bare ground and exotic species. We averaged vegetation characteristics across quadrats for each nest during analysis. Our vegetation sampling protocol was adapted from Wiens [49].

### 2.3. Statistical analysis

#### 2.3.1. Cowbird relative abundance

We used naive (unadjusted) estimates of brown-headed cowbird relative abundance for several reasons: (i) our research has demonstrated that detectability of brown-headed cowbirds in this ecosystem is high (perceptibility = 0.91, CI = 0.85–0.97; dependent double-observer sampling, *n* = 52, 3.2 ha plots); (ii) other researchers have demonstrated that unadjusted counts are appropriate with optimal indices of relative abundance of songbirds in northern mixed-grass prairies [50]; and because (iii) assumptions of distance sampling and removal sampling could not be met with our data [51], and not meeting these assumptions increases, rather than decreases, index bias (e.g. [52,53]). Further, noise from infrastructure does not alter the detectability of bird songs in this study area [44]. Therefore, we concluded that counts of cowbirds within each plot were a reasonable index of cowbird relative abundance, although they should not be considered an exact estimate of the population density [54].

All statistical analyses were conducted in program R [55]. Significance was evaluated using an *α*-value of 0.10 because we wanted to reduce the risk of making a Type II error, which results in significant problems in conservation biology [56]. Therefore, we calculated upper and lower 90% confidence intervals, and also reported standard errors (SEs) as an index of variation of parameter estimates. We used standard deviation (s.d.) to describe variation of the data when summarizing brown-headed cowbird relative abundances in specific treatments. We used generalized linear mixed models (GLMMs) [57] from the lme4 package [58] to model cowbird relative abundance. To determine if sites with oil and gas infrastructure had higher cowbird relative abundances, we first assessed effects of infrastructure at the site scale by including site type (infrastructure or control) and year as fixed effects, and by including site as a random effect in this model. For this analysis, the number of cowbirds observed within each transect was summed to provide an index of relative abundance at the scale of the site. We used Moran's I to test whether trends were spatially correlated among sites. As we found no evidence of spatial autocorrelation (−0.17 < Moran's *I* < 0.15; no pattern with distance class), we concluded that we could treat sites as independent in our analyses.

We then assessed whether relative abundances of cowbirds were higher near perch sites and roads. The number of cowbirds was counted within each of eight 100 × 100 m bins along each transect. We calculated the distance from the centre of each bin to the nearest perch (fence, power line or infrastructure) and to the nearest road. In this model, we examined cowbird relative abundance per bin relative to the fixed variables log (distance to nearest perch), log (distance to nearest road) and year, and included bin and site as random effects. We chose to use log (distance) because it performed better than distances calculated using an arithmetic scale with respect to model fit (ΔAIC_arithmetic_ 0.7–2.3).

We used information-theoretic and frequentist approaches to our analyses, each for different reasons. We used Akaike's Information Criterion (AIC) [59] for two purposes: first, to determine whether a random variable (site) and nuisance variables improved model fit and should be included in the above models, and second, to describe relative model fit (see below). For all other aspects of statistical analyses, in which we tested the effects of the fixed variables around which we designed our study, we adhered to a frequentist approach [60].

#### 2.3.2. Brood parasitism

We modelled probability of brood parasitism of Savannah sparrow nests using generalized linear modelling (GLM) and GLMM regression, accomplished using the lme4 package [58]. We constructed three models to analyse rates of brood parasitism of Savannah sparrow nests. We used AIC [59] to assess whether year, Julian date or a quadratic Julian-date term should be included in each of our three global models. In each case, only year improved model fit and was included. The inclusion of random effect site was also assessed in the same manner, and only improved model fit in our first model, thus the second and third models did not include a random effect and were run as GLM regressions. The first model assessed the risk of parasitism at the site scale and included the variables year, site type (control or infrastructure) and the random effect site. The second model was used to assess effects of the presence of perch sites and roads on brood parasitism by cowbirds. This model included the independent variables year, distance to nearest perch (i.e. structure, fence or power transmission or distribution line) and distance to nearest road. Finally, the third model examined the effect of vegetation characteristics on the probability of brood parasitism and included the independent variables year, stem density, stem height, litter depth, bare ground, exotic vegetation and shrub. We chose to keep these models separate as they each tested distinct hypotheses; this also allowed us to minimize collinearity among variables and to avoid over-parametrizing models.

#### 2.3.3. Nesting success

To determine if cowbird parasitism negatively impacted the survival of Savannah sparrow nests, we modelled the probability of nesting success using GLMM logistic exposure models [61]. Our nesting-success model included the fixed variables year, site type, parasitism status (presence or absence of cowbird eggs and/or nestlings) and the random effect site. Vegetation variables were not included in the survival model because they did not influence nesting success at our study sites [46].

#### 2.3.4. Model fit

Although there have been recent attempts to develop goodness of fit methods for mixed-effects models [62], there is currently no widely accepted approach, and available methods are not appropriate for both logistic exposure models and typical GLMMs. For consistency among our diverse statistical models, we used AIC [59] as a relative measure of model fit to assess whether the inclusion of independent variables improved model fit in comparison with null models.

## 3. Results

### 3.1. Cowbird relative abundance

**Figure 1.**
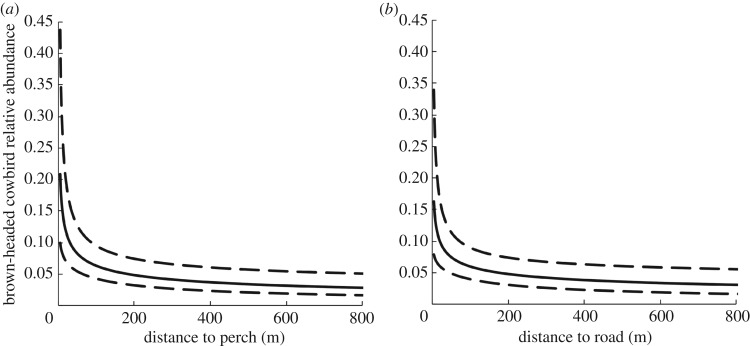
Brown-headed cowbird relative abundance per hectare near Brooks, AB during the 2013 and 2014 breeding seasons. Dashed lines denote upper and lower 90% confidence intervals. Distances were derived from calculating the inverse log of the model output. (*a*) Relative abundance as a function of distance to perch (power transmission or distribution lines, fences or energy infrastructure). (*b*) Relative abundance as a function of distances to road.

Cowbird relative abundance at infrastructure sites was 2.295/8 ha (s.d. = 0.433), approximately four times greater than at control sites, 0.519/8 ha (s.d. = 0.511) (*β* = 2.058, s.e. = 0.659, *p* =  < 0.001, model fit: ΔAIC_null_ = 6.2). From 2013 to 2014, cowbird relative abundance nearly doubled across both infrastructure and control sites, increasing by 99%, from 1.140 (s.d. = 0.421) to 2.264 (s.d. = 0.421) (*β* = 1.314, s.e. = 0.498, *p* = 0.008, model fit: ΔAIC_null_ = 27.9). Cowbird relative abundance increased significantly with proximity to both nearest perch (*β* = −0.380, s.e. = 0.120, *p* = 0.002, model fit: ΔAIC_null_ = 24.3) (figure 1*a*) and nearest road (*β* = −0.322, s.e. = 0.121, *p* = 0.008, model fit: ΔAIC_null_ = 21.0) (figure 1*b*).

### 3.2. Brood parasitism and nesting success

**Figure 2.**
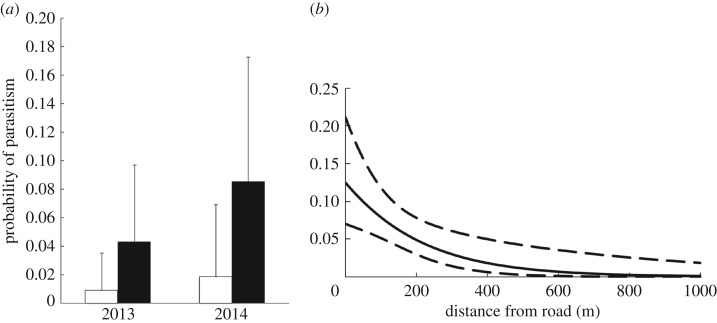
(*a*) Predicted parasitism rates of Savannah sparrow nests at control sites (white) and infrastructure sites (black) around Brooks, AB during 2013 and 2014 breeding seasons. Error bars denote upper 90% confidence intervals. (*b*) Predicted parasitism rates of Savannah sparrow nests around Brooks, AB during 2013 and 2014 breeding seasons as a function of distance to the nearest road. Dashed lines denote upper and lower 90% confidence intervals.

We found and monitored 427 Savannah sparrow nests during the 2013 and 2014 breeding seasons. On average, Savannah sparrow nests were found 367 m from infrastructure (range: 4–2361 m), 169 m from roads (range: 0–844 m) and 181 m from potential cowbird perches (range: 0–952 m). The probability of cowbird parasitism was significantly higher in 2014 (brown-headed cowbird relative abundance was also higher in 2014), compared with 2013 (*β* = 0.729, s.e. = 0.856, *p* = 0.058, model fit: ΔAIC_null_ = 2.1). Averaged across all sites, the overall probability of parasitism was 3% in 2013 and 5% in 2014. However, these averages are deceptive, as 92% of parasitism occurred at infrastructure sites, and parasitism was negligible (less than 2%) at control sites. The average probability of brood parasitism across years was four times greater at infrastructure sites relative to controls (*β* = 1.618, s.e. = 0.816, *p* = 0.048, model fit: ΔAIC_null_ = 2.1) (figure 2*a*). The probability of brood parasitism also increased with proximity to roads (*β* = −0.005, s.e. = 0.002, *p* = 0.018, model fit: ΔAIC_null_ = 11.8) (figure 2*b*), with increasing vegetation height (*β* = 8.775, s.e. = 3.088, *p* = 0.004, model fit: ΔAIC_null_ = 7.6) and with increasing cover of exotic vegetation (*β* = 0.017, s.e. = 0.007, *p* = 0.020, model fit: ΔAIC_null_ = 7.6).

**Figure 3.**
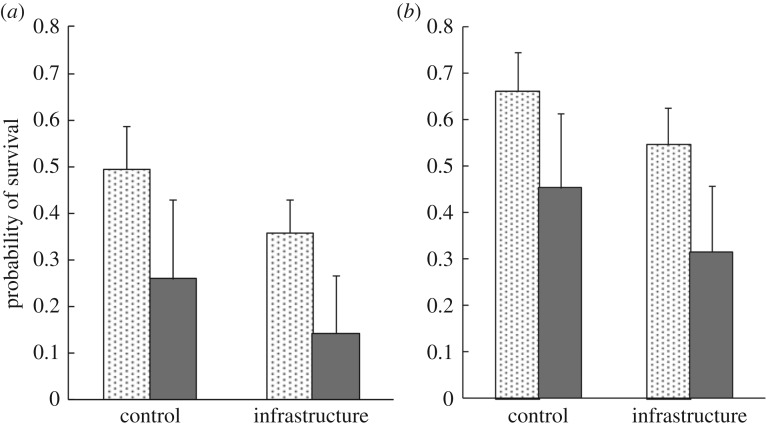
Predicted nest survival of un-parasitized (shaded) and parasitized (grey) Savannah sparrow nests around Brooks, AB. Errors bars denote upper 90% confidence intervals. (*a*) Nest survival during the 2013 breeding season at control and energy-infrastructure sites. (*b*) Nest survival during the 2014 breeding season at control and energy-infrastructure sites.

Nesting success was significantly higher in 2014 than in 2013 (*β* = 0.546, s.e. = 0.163, *p* = 0.001, model fit: ΔAIC_null_ = 7.3) and was significantly lower at infrastructure sites relative to controls (*β* = −0.382, s.e. = 0.198, *p* = 0.052, model fit: ΔAIC_null_ = 7.3). Parasitized nests had a 22% lower probability of survival than un-parasitized nests (*β* = −0.663, s.e. = 0.239, *p* = 0.005, model fit: ΔAIC_null_ = 7.3) (figure 3*a*,*b*). Additionally, successful parasitized nests (2.1 ± 1.1; *n* = 11) fledged 30% fewer host young on average than successful un-parasitized nests (3.3 ± 1.2; *n* = 223). The effects of oil and gas infrastructure on the nesting success of grassland songbirds are examined in detail in a separate study [46].

## 4. Discussion

Our models supported the hypothesis that brown-headed cowbirds are attracted to energy-infrastructure features, resulting in higher brood parasitism rates and lower productivity of hosts. Increases in relative abundance of cowbirds at infrastructure sites were probably a consequence of cowbirds' preference for edge habitat [9] and attraction to features such as perches [13] and roads [63]. This suggests that attraction of cowbirds to energy infrastructure contributes to negative effects of anthropogenic development on grassland songbird populations.

Although the overall parasitism rate across our study area was relatively low, the magnitude of the effect of wells and associated infrastructure on increasing brood parasitism rates was very large. The observed increased brood parasitism rates at sites containing energy infrastructure are consistent with a number of studies that have recorded increased cowbird parasitism of songbird nests near edges (e.g. [11,12,64]) and perches [14,65,66], and in areas of anthropogenic development [67–69]. However, our results are in contrast to a similar study that found no increase in brood parasitism rates on grassland songbirds nesting at oil infrastructure sites despite higher cowbird densities at these locations [70]. One possible explanation for this discrepancy is that our study focused only on the parasitism of Savannah sparrow nests, whereas Ludlow *et al*. [70] combined nests of multiple species in their brood parasitism models, a necessity due to their lower sample sizes. These factors might have resulted in lower power to detect effects on brood parasitism rates. Grassland songbirds differ in their behavioural responses to parasitism, as well as parasitism rates [31]; therefore, differences among species could have masked effects.

In our study, both cowbird density and brood parasitism rates also increased with proximity to roads. Roads are linear features and can act as edges affecting nest density and survival [71], as well as songbird abundance [63,72]. There is broad support demonstrating the positive effect of edges on cowbird brood parasitism in grassland systems (e.g. [11,25,73]). Edge effects created by roads may contribute to increased brood parasitism rates at infrastructure sites. However, the exact mechanism by which cowbirds might benefit from roads is unclear. It is typically argued that cowbirds are attracted to forest edges because of changes in host density and diversity [74], availability of perches [20,25] and a preference for forest-nesting host species [75,76]. Although some roads were associated with fence lines in our study area, this was not always the case, and the other mechanisms proposed seem unlikely to explain distributions of cowbirds in our study area. Cowbirds may simply be attracted to heterogeneous landscapes [9,69]. Roads can have significant ecological effects and may alter vegetation structure and species composition [77,78]. Dirt roads may also create suitable foraging habitat for cowbirds, which feed mainly on insects and seeds found in sparse vegetation or bare ground [79].

Although cowbird relative abundances were higher near perch sites in our study area, our results were not consistent with the perch availability hypothesis [9], as there was no relationship between distance to perch and frequency of brood parasitism. This may be because cowbirds are highly mobile and can travel up to 12 km between foraging habitat and host breeding grounds [80]. Consequently, cowbird parasitism often varies more with variables at the landscape scale than with those at the local scale, and thus edge effects related to brood parasitism may be apparent only at large spatial scales [9,81]. Increased frequency of parasitism at our infrastructure sites may be driven by increased cowbird relative abundance at the landscape level as a result of attraction to anthropogenic edges and perches introduced by infrastructure. Research suggests that brood parasitism rates are often determined by regional cowbird densities [82,83], and that both cowbird densities and parasitism frequencies are higher in fragmented and developed landscapes where edges are numerous (e.g. [67,81,84]).

Brood parasitism can have a multitude of negative effects on host survival and productivity in songbirds [85]. In our study area, the probability of nest survival was considerably reduced in parasitized nests (see also [30]). In addition to increased probability of abandonment [18] and increased hatching failure [85], parasitized nests often suffer higher predation rates [21], possibly because they are more likely to be discovered by predators as a consequence of loud cowbird nestling begging [86]. Songbird host young may also be outcompeted by larger and more aggressive cowbird nestlings [87], and the need to increase provisioning rates to meet higher cowbird nestling energetic demands reduces adult host fitness [88]. Given that parasitized Savannah sparrow nests at our study sites suffered both decreased survival and productivity, increased parasitism pressure as a consequence of oil wells and natural gas compressor stations may have a reproductive cost for regional populations of this species.

## 5. Management implications

Our results demonstrate that the presence of energy infrastructure has the potential to increase both cowbird relative abundance and brood parasitism rates in mixed-grass prairie habitat. These increases are most probably driven by increased perch availability, roads and edge habitat introduced to the landscape by oil wells and natural gas compressor stations. Given known relationships between both brood parasitism rates and nesting success relative to population trends of hosts [89–91], increasing risk of brood parasitism may be one mechanism that has contributed to negative population trends in grassland songbirds. With this in mind, managers should attempt to minimize the extent of both perch availability and roads associated with lease sites. This could be accomplished by burying power distribution lines (thus reducing perch availability), using small sections of technical fencing to exclude cattle from the immediate area around infrastructure in place of traditional fencing around the entire lease areas (which provides more perch sites) and drilling new wells horizontally or directionally at pre-existing well pads [92]. This last measure would not only reduce the number of surface structures on the landscape, but could also limit the need for new access roads. Similarly, use of multi-bore wells in place of numerous single-bore wells has also been shown to reduce the spatial impact of infrastructure on grassland songbirds [93]. Our findings suggest that oil and gas infrastructure may indirectly contribute to habitat degradation for grassland songbirds at spatial scales that extend beyond the immediate areas of disturbance.

## Data accessibility

All primary data collected and analysed for this study have been deposited with Dryad and can be accessed at: http://dx.doi.org/10.5061/dryad.d1h97 [94].
